# The Association Between Contentment and Depressive Symptoms: Results From Three Panel Studies

**DOI:** 10.1002/jclp.70082

**Published:** 2026-02-16

**Authors:** Shuo Yan, Nathaniel S. Eckland, Howard Berenbaum

**Affiliations:** ^1^ Department of Psychology University of Illinois at Urbana‐Champaign Champaign Illinois USA

**Keywords:** cheer, contentment, depression, discrete emotions, tranquility

## Abstract

We examined whether contentment was associated with depressive symptoms at both between‐ and within‐person levels, both concurrently and prospectively. We examined our hypotheses using random‐intercept cross‐lagged panel models (RI‐CLPM) that computed the associations between contentment and depressive symptoms, treating tranquility and cheer as covariates, with three sets of data: three waves of the Health and Retirement Study (HRS; *n* = 27,947), the Midlife in the United States (MIDUS) Refresher daily diary study (*n* = 782), and a daily diary study with college students (*n* = 278). For the between‐person and concurrent within‐person associations, in all three samples, contentment was associated with depressive symptoms, even when considering tranquility and cheer. Likewise, for the prospective associations, only contentment predicted subsequent depressive symptoms in two of the three samples (the HRS and the MIDUS samples). We discuss implications for studying the etiology and treatment of depression.

## 1 Introduction

The diminished ability to experience pleasure is a common feature of depressive symptoms (e.g., Berenbaum and Oltmanns 1992; Fawcett et al. 1983; Pizzagalli et al. 2008). Even though researchers have distinguished among a variety of pleasurable emotions, such as contentment, tranquility, and cheer (e.g., Berenbaum et al. 2016; Shiota et al. 2006), past research examining pleasure deficits and depressive symptoms has focused almost entirely on global positive affect. Given that different pleasurable emotions, such as contentment and tranquility, differ in how they are elicited and what they are associated with (e.g., Berenbaum et al. 2019), we expect them to also differ in how they are associated with depressive symptoms. This possibility is potentially quite important for understanding the etiology, experience, and treatment of depressive symptoms, and is what motivated the present research.

Eckland et al. (2021) hypothesized that low contentment would be an important risk factor for depression symptoms. Specifically, consistent with the vulnerability model Beck (1976), low contentment would be a risk factor for depressive symptoms, not the other way around (i.e., scar model; Lewinsohn et al. 1981). The hypothesis was based on previous work suggesting that contentment, but not tranquility or cheer, is elicited by acceptance of one's current status and a sense of completeness (Cordaro et al. 2016), as well as evidence that contentment is more strongly (positively) associated with life satisfaction than are tranquility and cheer (Berenbaum et al. 2013). These findings led Eckland et al. (2021) to posit that diminished contentment might lead to depressive symptoms because diminished contentment may lead to depressogenic cognitive distortions, such as viewing themselves as failures or worthless (Beck 1976). As had been predicted, diminished contentment has been found to be associated with increased depressive symptoms, with the associations typically being strong (Castro et al. 2024; Cordaro et al. 2021; DeHart et al. 2025; Eckland et al. 2021, 2022).

Although tranquility and cheer are highly correlated with contentment, the three emotions differ in various ways. Tranquility is similar to contentment in that it is also a low‐arousal pleasurable emotion that occupies essentially the same location on the affective circumplex as contentment (Russell 1980). Contentment is elicited by a sense of completeness (Berenbaum et al. 2019; Cordaro et al. 2016) and encourages the savoring of one's life circumstances and recent successes (Fredrickson 2001). In contrast, tranquility describes a state of being at peace, regardless of goal attainment (Berenbaum et al. 2016, 2019), and a sense of certainty about one's future (Ellsworth and Smith 1988); given that tranquility reflects a state of pleasant inactivity of body and mind (Cordaro et al. 2016), it likely promotes rest and recuperation. Previous research has found that contentment is positively associated with mastery activities and is not associated with spiritual activities, whereas tranquility is negatively associated with mastery activities but is positively associated with spiritual activities (Berenbaum et al. 2019). Furthermore, contentment is associated with increased levels of extraversion, conscientiousness, and meeting the need for competence, whereas tranquility is characterized by low levels of extraversion and meeting the need for predictability (Berenbaum et al. 2016; Yan et al. 2024). In contrast to contentment and tranquility, cheer is a high‐arousal form of pleasurable emotion that is usually described as joy or happiness (Lazarus 1991; Shiota et al. 2006); it is strongly associated with extraversion, social activities (Berenbaum 2002;1 Berenbaum et al. 2016) and affiliation satisfaction (Yan et al. 2024).

The previous studies examining the relation between contentment and depressive symptoms were all cross‐sectional and examined the relation at the between‐person level. We expanded previous between‐person, cross‐sectional studies examining the relation between contentment and depressive symptoms in two ways. First, the previous between‐person studies only answered the question “who is more likely to be depressed:” whether those people who are less contented are more likely to have higher levels of depressive symptoms than are those people who are more contented. The current research also examined the within‐person associations to answer the question “when is someone more likely to be depressed:” whether, on those occasions when a person is less contented, that same person experiences more depressive symptoms than on those occasions when that person is more contented. Finding an association between contentment and depressive symptoms at the within‐person level renders less plausible the alternative explanation that the association between contentment and depressive symptoms is attributable to a third between‐person variable, such as emotion differentiation abilities. Associations at either level would support our hypothesis; however, convergent results at both the between‐ and within‐person levels would provide more robust support. Second, because of their cross‐sectional nature, previous studies were unable to draw conclusions regarding temporal precedence. The present research examined whether contentment would be prospectively associated with depressive symptoms and not the other way around.

We tested our hypotheses using three samples, two of which are large, publicly available data sets (i.e., the Health and Retirement Study [HRS] and the MIDUS Refresher daily diary study, and we collected the third sample from college students. Using the HRS sample, we examined how contentment, tranquility, and cheer experienced in the past 30 days were associated with depressive symptoms over a 2‐week period measured concurrently and 4 years later. Using the MIDUS and the college student samples, we examined how contentment, tranquility, and cheer on a given day were associated with daily experiences of depressive symptoms on the same day and the following day. Convergent results for contentment being particularly strongly (negatively) associated with depressive symptoms across different samples, measures, time scales (i.e., 2 weeks vs. 1 day) and time intervals across waves (1 day vs. 4 years) would provide strong evidence for such a link because it would render less plausible the possibility that the association is limited to the idiosyncratic methods/measures of a single study. To address the alternative explanation (i.e., the scar model), we also examined whether depressive symptoms would predict later contentment.

## 2 Methods

### 2.1 HRS Sample

#### 2.1.1 Participants

The HRS is sponsored by the National Institute on Aging and is conducted by the University of Michigan (https://hrs.isr.umich.edu). We used data from 2008, 2012, and 2016 because measures of pleasurable emotions and depressive symptoms were available. Among participants included in our sample (i.e., who had depressive symptoms and pleasurable emotion data available; total *n* = 27,947), 55.8% were female, 44.2% were male;^2^ 70.6% identified their race as White/Caucasian, 18.4% Black/African American, 8.1% reported a race other than Black or White which was reported as one category (including American Indian, Alaskan Native, Asian, Native Hawaiian, Pacific Islander, or another race), and 2.8% did not report their race; 12% reported Hispanic or Latinx ethnicity. Their ages ranged from 20 to 98 (mean = 67.5; SD = 16.5).

#### 2.1.2 Measures

##### 2.1.2.1 Pleasurable Emotions

Participants rated the extent to which they experienced a list of affective states over the past 30 days. Items were rated on a 5‐point scale (1 = *Very much to* 5 = *Not at all*) corresponding to how much the individual felt that emotion in the past 30 days. We reverse‐coded items so higher values indicated experiencing the emotion more often. We used the item “happy” to represent cheer, “content” to represent contentment, and “calm” to represent tranquility. These items have been used in previous research that showed high item loadings in confirmatory factor analysis, high average inter‐item correlation, and evidence for reliability and convergent and discriminant validity.

##### 2.1.2.2 Depressive Symptoms

Depressive symptoms were measured using the Composite International Diagnostic Interview‐Short Form (CIDI‐SF; Kessler et al. 1998). The short form has shown high agreement with the original CIDI (Kessler et al. 1998; Levinson et al. 2017; Patten et al. 2000), as well as high reliability and high item loadings in confirmatory factor analysis (Dang et al. 2020; Gigantesco and Morosini 2008). Participants were asked two screening questions about depressed mood and anhedonia for a 2‐week period over the past year. Participants who endorsed either of those symptoms in the past year were asked follow‐up questions about the remaining symptoms of Major Depressive Disorder. Endorsed symptoms were summed, creating a scale of 0 (no symptoms endorsed) to 8 (all symptoms endorsed). At Wave 1, 16,077 participants were asked about depressive symptoms—13.9% (*n* = 2229) of participants reported at least one depressive symptom, and the responses of 5.4% (*n* = 644) provided were suggested that they likely met criteria for a major depressive episode (i.e., they endorsed at least five of the MDE criteria). At Wave 2, 19,406 participants were asked about depressive symptoms—14.9% (*n* = 2887) of participants reported at least one depressive symptom, and 5.9% (*n* = 1019) likely met criteria for a major depressive disorder. At Wave 3, 19966 participants were asked about depressive symptoms—0.7% (*n* = 3743) of participants reported at least one depressive symptom, and 7.4% (*n* = 1473) likely met criteria for a major depressive disorder.

### 2.2 MIDUS Sample

#### 2.2.1 Participants

Data were drawn from the MIDUS Refresher daily diary study^3^ conducted between the second and third waves of the MIDUS (https://doi.org/10.3886/ICPSR37083.v1). In total, 782 people (55.6% female; 44.4% male;^4^ 84.1% White; 6.8% Black; 1.4% Native American; 1.1% Asian; 6.6% reported a race other than those previously listed; 5.4% Latino/Latina ethnicity) participated in the daily diary study. Their ages ranged from 25 to 75 (mean = 47.9 years; SD = 12.7 years). Participants responded to 93.7% of daily surveys.

#### 2.2.2 Measures

##### 2.2.2.1 Pleasurable Emotions

Each night for 8 consecutive days, participants rated the extent to which they experienced a list of affective states over the course of the day. Items were rated on a 5‐point scale (0 = *None of the time* to 4 = *All of the time*) corresponding to how much the individual felt the emotion that day. We recoded items to be on a 1–5 scale to be consistent with the HRS sample described above. We used the item “cheerful” to represent cheer, “satisfied” to represent contentment, and “calm” to represent tranquility. Similar to the items used in the HRS sample, these items have shown evidence for reliability, structural validity, and convergent and discriminant validity in previous research (Berenbaum et al. 2016, 2019; DeHart et al. 2025; Yan et al. 2024).

##### 2.2.2.2 Depressive Symptoms

Four questions were from the Kessler Psychometric Distress Scale (K‐10; Brooks et al. 2006; Kessler et al. 2003) to measure depressive symptoms. Previous studies have shown that these items have high item loadings on a depression latent variable (Brooks et al. 2006; Pereira et al. 2019) and that the subscale showed high intraclass correlations (Cleofas 2024; Vissoci et al. 2018). Moreover, the items were identical or quite similar to items in popular depression questionnaires, such as the Center for Epidemiological Studies – Depression Scale (CES‐D; Radloff 1977) and the Beck Depression Inventory – II (BDI; Beck et al. 2011). These four items, which follow, were used to measure daily levels of depressive symptoms: “felt worthless,” “felt so sad that nothing would cheer you up,” “felt like everything was an effort,” and “felt hopeless.” Items were rated on a 5‐point scale (0 = *None of the time* to 4 = *All of the time*) corresponding to how the individual felt that day. Within‐ and between‐person reliabilities were acceptable (*ω*_*within*_ = 0.60, *ω*_*between*_ = 0.82).

### 2.3 College Student Sample

#### 2.3.1 Participants

Out of 366 participants who began the study, 278 (65.5% female or primarily feminine, 33.1% male or primarily masculine, 0.4% reported being neither male nor female, 0.4% reported being both male and female, and 0.4% reported “don't know”) provided sufficient data (see details below) to be included in analyses. They ranged in ages from 18 to 28 (M = 18.9, SD = 1.3). 52.2% reported being White/European American, 28.8% reported being Asian/Asian American, 4.3% reported being Black or African American, 5.0% reported being multiracial, and 9.7% reported none of the above. 18.0% reported being of Hispanic or Latin ethnicity. All participants provided informed consent and received course credit for their participation. All measures and procedures were approved by the IRB at the University of Illinois at Urbana‐Champaign prior to the beginning of data collection. The dataset is available in Open Science Framework, https://osf.io/mu6yd/?view_only=1e8e1cb50dae4b4198b8cd52120d5ff2.

#### 2.3.2 Procedures

All participants were recruited from the University of Illinois at Urbana‐Champaign Psychology Department subject pool. Before registering for any studies, the participants answered a set of pre‐screening questions, which contained the Patient Health Questionnaire (PHQ‐9; Kroenke et al. 2001) that measures depressive symptoms. We over‐sampled participants who tended to be the most depressed by encouraging those who scored in the top 15% range of PHQ‐9 to participate through email advertisements (28.9%, *n* = 77; the pre‐screening information for five participants was lost). 26.8% of participants (*N* = 121) had PHQ‐9 scores greater than 10, which is the recommended cutoff for a likely major depressive episode (Manea et al. 2015).

Participants received a link to a daily diary survey (described below) once every day for 7 consecutive days. Participants completed all daily diaries remotely on their own devices. All daily diaries were hosted on the Qualtrics website and were formatted so that they could be accessed through computers and mobile devices. All seven daily dairies were identical. The links were sent at 5:00 p.m., which was considered the end of the school day. The links expired at 4:00 a.m. the next day, which was considered late enough so that the participants had enough time to complete the daily diary before bedtime, but too early for any morning activities. Participants were instructed to complete at least five daily diaries to receive course credits. Participants who completed fewer than five daily diaries over the 7‐day protocol were excluded from analyses. The rationale for requesting five daily surveys was to get sufficient data to measure within‐person fluctuations and was not too many for participants to lose incentives for one course credit.

#### 2.3.3 Measures^5^

##### 2.3.3.1 Daily Depressive Symptoms

Prompted by “please indicate the degree to which each of the following was true for you today,” participants answered seven items on a 5‐point scale (1 = *Not at all true* to 5 = *Completely true*; *ω*_*within*_ = 0.63, *ω*_*between*_ = 0.93) regarding their daily depressive feelings using a revised version of the Patient Health Questionnaire (PHQ‐9; Kroenke et al. 2001). We did not include the questions regarding suicidality, psychomotor agitation, psychomotor retardation, and concentration.^6^ The items used in the current study have shown high factor loadings (Bianchi et al. 2022; Vu et al. 2022). The daily depressive symptom scores were computed as the mean of the items.

##### 2.3.3.2 Daily Emotions

Prompted by “indicate the extent to which you felt this way today,” participants were asked to rate the degree to which they felt contented, tranquil, and cheerful each day on a 5‐point scale (1 = *Very slightly or not at all* to 5 = *Extremely*). We measured contentment, tranquility, and cheer using items from our previous research (Berenbaum et al. 2013, 2016, 2019; Sun et al. Unpublished manuscript) that showed high item loadings, intraclass correlations, test‐retest reliability, and convergent and discriminant validity. Cheer was measured using “upbeat,” “fun,” and “cheerful” (*ω*_*within*_ = 0.78, *ω*_*between*_ = 0.97); contentment was measured using “a sense of completeness,” “satisfied with what I have,” and “fulfilled” (*ω*_*within*_ = 0.69, *ω*_*between*_ = 0.97); and tranquility was measured using “tranquil,” “calm,” and “at ease” (*ω*_*within*_ = 0.68, *ω*_*between*_ = 0.95). All three pleasurable emotion scores were computed using the mean of the items, respectively.^7^

### 2.4 Date Analysis

Following the recommendations of Leys et al. (2013) we winsorized all scores using the mean absolute deviation (MAD) with a threshold of 2.5 and a constant of 1.4826 prior to conducting the main analyses.

To examine our hypotheses, we constructed random‐intercept cross‐lagged panel models (RI‐CLPM; Hamaker et al. 2015). RI‐CLPMs allow us to decompose variances into between‐ and within‐person components and optimally model within‐person changes. We examined how contentment, tranquility, and cheerfulness were each associated with concurrent and prospective depressive symptoms at the between‐ and within‐person levels, accounting for the within‐person autoregressive effects and regression towards the mean (e.g., Usami 2021; Zainal and Newman 2021). While depressive symptom scores, contentment, tranquility, and cheer were entered into the models simultaneously, the focus was on the concurrent and prospective paths between contentment and depressive symptoms, treating tranquility and cheer as covariates. Missing data was addressed using full information maximum likelihood (FIML) estimation. We constructed all models using the lavaan package (Rosseel 2012) in R version 4.2.3. The HRS model examined within‐ and between‐year associations (across 4‐year periods), whereas the MIDUS and the college student sample model examined within‐ and between‐day associations between the pleasurable emotions and depressive symptoms. We were particularly interested in the cross‐paths from the pleasurable emotions to later depressive symptoms. We also examined the prospective paths from depressive symptoms to pleasurable emotions. To allow for fewer parameters to be estimated overall and for greater parsimony in model interpretation, we imposed stationarity on the model (i.e., corresponding cross‐paths and autoregressive paths were constrained to be equal). Additionally, the corresponding concurrent correlations between variables were also constrained to be equal across waves. We computed standardized model estimates and interpreted the magnitudes of the between‐person and concurrent within‐person effects using cutoffs of 0.1, 0.2, and 0.3 (for small, medium, and large, effects, respectively) as established by Brydges (2019) and Gignac and Szodorai (2016). We interpreted the magnitudes of the cross‐lagged effects using cutoffs of 0.03, 0.07, and 0.11, as established by Orth et al. (2024).

## 3 Results

Descriptive statistics are presented in Table S1 in the Supporting Information. The model fit indices for the HRS sample (*χ* ^*2*^ [34] =65.23, *p* = 0.001, CFI = 0.999, RMSEA = 0.006, SRMR = 0.011, the MIDUS sample (*χ* ^*2*^ [464] = 772.25, *p* < 0.01, CFI = 0.983, RMSEA = 0.029, SRMR = 0.039), and the college student sample (*χ* ^*2*^ [346] = 444.44, *p* ≤ 0.001, CFI = 0.982, RMSEA = 0.042, SRMR = 0.045) indicate that the three models were good fits for the three samples, respectively.^8^

### 3.1 Between‐Person Associations

The between‐person associations between pleasurable emotions and depressive symptoms are shown in Table 1. Contentment was significantly negatively associated with depressive symptoms in all three samples, even when taking into account tranquility and cheer, with effect sizes being medium to large. Tranquility and cheer were also significantly associated with depressive symptoms in all three samples, with effect sizes similar to those found for contentment.

**Table 1:** Between‐person, concurrent within‐person, and prospective within‐person associations between pleasurable emotions and subsequent depressive symptoms.

	*HRS* (*n* = 27,947)				*MIDUS* (*n* = 782)				*College Student* (*n* = 266)			
	Estimate	SE	*z*	*p*	Estimate	SE	*z*	*p*	Estimate	SE	*z*	*p*
	*Between‐Person Associations*											
Contentment	−0.17	0.01	−13.52	< 0.001	−0.26	0.03	−10.39	< 0.001	−0.28	0.05	−6.19	< 0.001
Tranquility	−0.18	0.01	−16.11	< 0.001	−0.23	0.02	−9.38	< 0.001	−0.25	0.04	−5.65	< 0.001
Cheer	−0.19	0.01	−16.83	< 0.001	−0.23	0.02	−9.36	< 0.001	−0.20	0.04	−4.95	< 0.001
	*Concurrent Within‐Person Associations*											
Contentment	−0.08	0.01	−8.24	< 0.001	−0.13	0.01	−19.29	< 0.001	−0.17	0.01	−13.25	< 0.001
Tranquility	−0.06	0.01	−6.72	< 0.001	−0.12	0.01	−17.47	< 0.001	−0.16	0.01	−11.34	< 0.001
Cheer	−0.06	0.01	−5.08	< 0.001	−0.12	0.01	−18.16	< 0.001	−0.20	0.02	−13.67	< 0.001
	*Prospective Within‐Person Associations*											
Contentment	−0.05	0.02	−2.48	0.01	−0.06	0.02	−2.82	0.005	0.04	0.05	0.74	0.46
Tranquility	−0.01	0.02	−0.44	0.66	0.01	0.02	0.31	0.76	−0.08	0.04	−1.76	0.08
Cheer	0.003	0.02	0.15	0.88	−0.01	0.02	−0.69	0.49	0.01	0.05	0.21	0.83

### 3.2 Concurrent Within‐Person Associations

The concurrent within‐person associations between pleasurable emotions and depressive symptoms are shown in in Table 1. Again, in all three samples, as expected, contentment was significantly negatively associated with depressive symptoms, even when taking into account tranquility and cheer, with effect sizes being in the small to medium range. Tranquility and cheer were also significantly associated with depressive symptoms in all three samples, with effect sizes similar to those found for contentment. Although contentment was more strongly associated with depressive symptoms than was tranquility in all three samples, and was more strongly associated with depressive symptoms than was cheer in two of the three samples, the differences in magnitudes were quite small.

### 3.3 Prospective Within‐Person Associations

The prospective associations between contentment and subsequent depressive symptoms are shown in Table 1. Our hypothesis that only contentment would be associated with prospective depressive symptoms was supported in two of the three samples, the HRS (*β* = −0.05, *p* = 0.01) and MIDUS samples (*β* = −0.06, *p* = 0.005), with associations in the small to medium range. In contrast, in the HRS and MIDUS samples, the prospective associations between the other two pleasurable emotions and depressive symptoms were negligible. In the college student sample, it is worth noting that only tranquility predicted next‐day depressive symptoms in the expected direction, but fell short of statistical significance (*β* = ‐0.08, *p* = 0.08). Of note, as shown in Table 2, higher depressive symptoms did not prospectively predict higher subsequent pleasurable emotions in any sample.

**Table 2:** Standardized coefficients for depressive symptoms predicting subsequent pleasurable emotions.

	*HRS* (*n* = 27,947)				*MIDUS* (*n* = 782)				*Daily Diary* (*n* = 266)			
	Estimate	SE	*z*	*p*	Estimate	SE	*z*	*p*	Estimate	SE	*z*	*p*
Contentment	−0.02	0.02	−0.92	0.36	0.01	0.02	0.81	0.42	0.03	0.04	0.75	0.45
Tranquility	0.01	0.02	0.64	0.53	0.01	‐0.02	1.59	0.64	0.04	0.05	0.95	0.34
Cheer	0.01	0.02	0.67	0.50	0.01	0.02	0.70	0.49	0.01	0.05	0.11	0.92

## 4 Discussion

The current study extended the previous examination of the between‐person, cross‐sectional associations between pleasurable emotions and depressive symptoms by also examining their prospective associations at the within‐person level. We replicated the finding that contentment continued to be negatively associated with depressive symptoms after taking into account the other two pleasurable emotions at the between‐person level (Eckland et al. 2021, Study 2), and extended this finding to the within‐person level. Extending the finding to the within‐person level is important because within‐person associations cannot be accounted for by between‐person individual difference variables, such as age or gender.

For concurrent associations, that contentment was strongly (negatively) associated with depressive symptoms at both the between‐ and within‐person levels, even when accounting for cheer and tranquility, provides robust support for diminished contentment being associated with depressive symptoms. Specifically, the results provided evidence that those people *who* are less contented experience higher levels of depressive symptoms than those *who* are more contented. In addition, *when* people are less contented, they experience higher levels of depressive symptoms than *when* they are more contented.

We found that diminished levels of contentment prospectively predicted higher levels of depressive symptoms in two of the three samples (the HRS and the MIDUS samples); in both of these samples, the prospective associations between the other two pleasurable emotions and depressive symptoms were negligible. Moreover, in the HRS and the MIDUS samples, the findings were consistent across two different time scales; in the HRS sample, pleasurable emotions and depressive symptoms were measured 4 years apart, whereas in the MIDUS sample, pleasurable emotions and depressive symptoms were measured daily. In addition, we found no evidence that depressive symptoms prospectively predict any of the pleasurable emotions examined. The results support the directionality from contentment to subsequent depressive symptoms, providing evidence for the vulnerability rather than the scar model.

Despite the evidence for the predictive association between low contentment and depressive symptoms, we do not yet know why this is the case. We hypothesize that the association between contentment and depressive symptoms, even when accounting for other pleasurable emotions, may be due to some characteristics about contentment that do not describe other pleasurable emotions. For example, a sense of competence and completeness, a theme of contentment (Berenbaum et al. 2013; Cordaro et al. 2021), may be particularly relevant to some depressogenic cognitive distortions (such as viewing themselves as failures or worthless, or a sense of helplessness and/or hopelessness) which makes people vulnerable to depressive symptoms (Harrison et al. 2022; Nolen‐Hoeksema et al. 1986). It will be important for future research to determine whether contentment contributes to the risk for depressive symptoms or whether it just happens to be associated with another variable that plays a causal role. Even if contentment plays a causal role influencing subsequent depressive symptoms, we do not yet know how this occurs and over what time period. Our results indicate that contentment predicts depressive symptoms over both very brief time frames (1 day) and over very long time frames (4 years). It is possible that diminished contentment contributes to vulnerability to depressive symptoms through its contributions to the depressogenic cognitive distortions. Such vulnerability may sometimes be sufficient to increase depressive symptoms the next day (Alloy et al. 1997; Ebrahimi et al. 2021). It is also possible that diminished contentment and depressogenic cognitive distortions form a positive feedback loop (i.e., diminished contentment contributes to depressogenic cognitions, and depressogenic cognitions contribute to diminished contentment). To the degree that diminished contentment and the cognitive vulnerabilities to which diminished contentment may contribute are temporally stable (and possibly even self‐perpetuating), one would expect diminished contentment at a single point in time to predict depressive symptoms years later.

Unlike the HRS and MIDUS samples, we did not find evidence of contentment prospectively predicting depressive symptoms in the college student sample. It is possible that, rather than contentment, tranquility is associated with subsequent depressive symptoms among young adults; in the college student sample, only tranquility predicted next‐day depressive symptoms in the expected direction but fell short of statistical significance. Perhaps tolerance of uncertainty, which differs by developmental stages (Schweizer et al. 2023), is more critical to the mental health outcomes of younger than older adults. Thus, among young adults, tranquility, which is strongly associated with a sense of certainty Ellsworth and Smith (1988) and predictability (Yan et al. 2024), may be more predictive of subsequent depressive symptoms than is contentment.

Additional research is needed to explore the association between contentment and other factors that have been found in past research to be associated with depressive symptoms, such as reward processing (Keren et al. 2018) and childhood trauma (e.g., Heim et al. 2008). To the extent that contentment and other depressive symptoms‐related factors are correlated, it would provide clues to the manner in which contentment and these other factors contribute to depressive symptoms (e.g., links between trauma and depressive symptoms possibly being mediated by contentment). On the other hand, to the extent that contentment and other depression‐related factors are not correlated, it would suggest the possibility of alternative, at least partially independent, pathways to depressive symptoms.

An additional important avenue for future research is exploring the potential role of contentment in the treatment of depressive symptoms. Behavioral activation for depressive symptoms involves encouraging people to engage in activities that contribute to pleasant emotions. Our results suggest that engaging in activities aimed specifically at eliciting contentment may have greater long‐term benefits than activities aimed at eliciting pleasant emotions in general, or emotions such as cheer. Interventions other than behavioral activation also have the potential to target diminished contentment. For example, interventions that target cognitions that promote contentment (e.g., the belief that one is whole or complete) and traditional cognitive therapies that target depressogenic cognitive distortions (which we hypothesize contribute to diminished contentment) may be useful for increasing levels of contentment and diminishing risk for depressive symptoms.

Two public data sets used to test our hypotheses were not collected with our hypotheses in mind. Consequently, in two of the three samples we examined, the three pleasurable emotions of interest in the current study (i.e., contentment, cheer, tranquility) were each measured using only a single self‐report item. Another limitation of the present research is that the samples were unlikely to include many participants with severe levels of depressive symptoms. Given that previous research in two clinical samples found that contentment was associated with depressive symptoms even after taking into account cheer and tranquility (Eckland et al. 2021), we expect the prospective association between contentment and depressive symptoms to be most clear when samples include reasonable representation at the high end of the depressive severity continuum; future research is needed to confirm that this is the case. Furthermore, for the HRS sample, depressive symptom severity was computed using the number of symptoms endorsed on the CIDI‐SF, and the CIDI was not designed to measure symptom severity.

Despite its limitations, the current study is the first to examine the within‐person and prospective associations between contentment and depressive symptoms. The current study adds to the evidence that contentment is associated with depressive symptoms cross‐sectionally, and is the first to provide evidence that increased contentment predicts increased subsequent depressive symptoms. These results suggest that studying specific pleasurable emotions, such as contentment, may provide information regarding the etiology and treatment of depressive symptoms that is not provided when measuring global positive affect alone.

## Supporting information


**Table S1:** Descriptive statistics of, and Pearson correlations between, wave‐aggregated scores. **Table S2:** Item loadings for the Weekly Averages of Emotion Items. **Table S3:** The associations between pleasant emotions and distress. **Table S4:** The associations between brokenness, exhaustion, and pleasurable emotions. **Table S5:** The associations between brokenness, exhaustion, and psychological distress. **Table S6:** The associations between contentment and depressive symptoms in the MIDUS sample, with and without the item “felt so sad that nothing would cheer you up”.

## Data Availability

The data that support the findings of this study are openly available in Daily Diary Study of Contentment and Depression at https://osf.io/mu6yd/?view_only=1e8e1cb50dae4b4198b8cd52120d5ff2.

## Endnotes

1 The Berenbaum (2002) study that found that cheerfulness was more strongly associated with social activities than was contentment did not examine tranquility.

2 Participants were asked to indicate whether they were male or female.

3 This was the only publicly available daily diary sample available when we began the study and the analyses were conducted.

4 Participants were asked to indicate whether they were male or female.

5 Several additional items measured worry, feelings of brokenness, and exhaustion, which were not the focus of the study and were not reported in the current manuscript. See section titled “Measures in the Daily Diary Study That Are Not Included in the Manuscript” in the Supporting Information.

6 We separated “sleeping too much” and “sleeping too little” into separate questions, and “eating too much” and “eating too little” into separate questions and took the higher value of each of the separated questions to calculate the depression scores.

7 We examined the factor structure of the weekly averages of emotion items using parallel analysis and exploratory factor analysis. Results are shown in the section titled “Factor Structure of the Daily Emotion Items” and Table S2 in the Supporting Information.

8 The results do not change when the item mentioning the word “cheer” is removed from the depressive symptoms measure; see Table S6 in the Supporting Information.
