# Supplementary material for: The Association Between Contentment and Depressive Symptoms: Results From Three Panel Studies
Source: J Clin Psychol. 2026 Feb 16;82(4):513–20. doi: 10.1002/jclp.70082 (PMC12965042; doi:10.1002/jclp.70082)
Supplement: Supplementary file 1 — Table S1: Descriptive statistics of, and Pearson correlations between, wave‐aggregated scores. Table S2: Item loadings for the Weekly Averages of Emotion Items. Table S3: The associations between pleasant emotions and distress. Table S4: The associations between brokenness, exhaustion, and pleasurable emotions. Table S5: The associations between brokenness, exhaustion, and psychological distress. Table S6: The associations between contentment and depressive symptoms in the MIDUS sample, with and without the item “felt so sad that nothing would cheer you up”. [file JCLP-82-513-s001.docx]

**Supporting Information**

**Table S1**

*Descriptive statistics of, and Pearson correlations between, wave-aggregated scores*

|  |  | Contentment | Tranquility | Cheer | Mean (SD) | Range |
| --- | --- | --- | --- | --- | --- | --- |
| HRS | |  |  |  |  |  |
|  | Depression | -.39*** | -.35*** | -.35*** | .1 (0.3) | [0, 3.1] |
|  | Contentment |  | .74*** | .66*** | 3.7 (0.8) | [1, 5] |
|  | Tranquility |  |  | .63*** | 3.7 (0.8) | [1, 5] |
|  | Cheer |  |  |  | 3.6 (0.8) | [1, 5] |
| MIDUS | |  |  |  |  |  |
|  | Depression | -.25*** | -.26*** | -.27*** | .6 (1.6) | [0, 8] |
|  | Contentment |  | .56*** | .66*** | 3.6 (1.1) | [1, 5] |
|  | Tranquility |  |  | .57*** | 3.6 (1.0) | [1, 5] |
|  | Cheer |  |  |  | 3.9 (1.0) | [1, 5] |
| Daily Diary Study | |  |  |  |  |  |
|  | Depression | -.48*** | -.45*** | -.42*** | 1.99 (0.72) | [1, 4.5] |
|  | Contentment |  | .78*** | .83*** | 3.14 (0.85) | [1, 5] |
|  | Tranquility |  |  | .73*** | 2.96 (0.74) | [1.06, 5] |
|  | Cheer |  |  |  | 2.95 (0.80) | [1, 5] |

*Note.* **p* < .05; ***p* < .01 ****p* < .001.

**Factor Structure of the Daily Emotion Items**

Parallel analysis (Horn, 1965) shows that the optimal number of factors is 3. We further examined the factor structure of the daily emotion items using exploratory factor analysis with maximum likelihood estimation. Based on the cutoff established by Hu & Bentler (1999), the model showed excellent fit (*χ^2^* [12] = 1.75, *p* = .09; CFI = .998; TLI = .994; RMSEA = .04). As shown in Table S2, item loadings of the daily emotion items replicated the previous results (e.g., Eckland et al., 2021; Sun et al., [Unpublished manuscript]), and suggested that contentment, tranquility, and cheer are distinct pleasurable emotions.

**Table S2**

*Item loadings for the Weekly Averages of Emotion Items*

|  | Contentment | Cheer | Tranquility |
| --- | --- | --- | --- |
| Fulfilled | **0.75** | 0.16 | 0.07 |
| Completeness | **0.94** | 0.02 | 0.00 |
| Satisfied | **0.84** | -0.06 | 0.01 |
| Tranquil | -0.12 | 0.07 | **0.84** |
| Calm | 0.03 | -0.03 | **0.91** |
| At ease | 0.14 | -0.01 | **0.82** |
| Fun | 0.01 | **0.92** | -0.01 |
| Cheerful | 0.07 | **0.86** | 0.02 |
| Upbeat | -0.04 | **0.91** | 0.01 |

**Measures in the Daily Diary Study That Are Not Included in the Manuscript**

In addition to using random-intercept cross-lagged panel models, we examined the associations between depression and the three pleasurable emotions (i.e., contentment, tranquility, and cheer) using Pearson correlations and linear regression at the between-person level and hierarchical linear models at the within-person level. Moreover, we examined how the three emotions were associated with worry, a feeling of brokenness, and exhaustion. The measures for worry, a feeling of brokenness, and exhaustion were described below. The results are shown in Tables S3, S4, and S5.

**Measures**

**Daily Worry.** Prompted by “please indicate the degree to which each of the following was true for you today,” participants answered 3 items (e.g., “today, I worry all the time”) on a 5-point scale (1 = *Not at all true* to 5 = *Completely true*; *ω_within_* = .84*; ω_within_* = .99) regarding their daily worry feelings using Penn State Worry Questionnaire – 3-item version (PSWQ-3; Kertz et al., 2004).

**Daily Feeling of Brokenness.** Prompted by “please indicate to which extant you have felt this way today,” participants answered 3 items, “broken”, “defective”, and “inadequate”, on a 5-point scale (1 = *Very slightly or not at all* to 5 = *Extremely*; *ω_within_* = .66*; ω_within_* = .96) regarding their daily feelings of brokenness using a subset of items from an unpublished scale (Castro et al., [Unpublished manuscript]).

**Daily Exhaustion**. Prompted by “please indicate to which extant you have felt this way today”, participants answered 3 items, “emotional exhaustion”, “mental exhaustion”, and “physical exhaustion”, on a 5-point scale (1 = *Very slightly or not at all* to 5 = *Extremely*; *ω_within_* = .70*; ω_within_* = .96).

**References**

Castro A.C. & Berenbaum H. [Unpublished manuscript]. *Illinois Inadequacy Scale* [Unpublished raw data]. Department of Psychology, University of Illinois at Urbana-Champaign.

Horn, J. L. (1965). A rationale and test for the number of factors in factor analysis. Psychometrika, 30(2), 179–185.

Hu, L.-t., & Bentler, P. M. (1999). Cutoff criteria for fit indexes in covariance structure analysis: Conventional criteria versus new alternatives. Structural Equation Modeling, 6(1), 1–55. [https://doi.org/10.1080/10705519909540118](https://psycnet.apa.org/doi/10.1080/10705519909540118)

Kertz, S. J., Lee, J., & Björgvinsson, T. (2014). Psychometric properties of abbreviated and ultra-brief versions of the Penn State Worry Questionnaire. *Psychological Assessment, 26*(4), 1146-1154. <https://doi.org/10.1037/a0037251>

**Table S3**

*The associations between pleasant emotions and distress*

|  |  |  | Depression |  |  | Worry |  |
| --- | --- | --- | --- | --- | --- | --- | --- |
|  |  | Cheer | Content | Tranquil | Cheer | Content | Tranquil |
| Between-person | |  |  |  |  |  |  |
|  | Individual | -.42*** | -.48*** | -.44*** | -.33*** | -.42*** | -.46*** |
|  | Simultaneous | -.02 | -.32** | -.18* | .15 | -.25* | -.38*** |
| Within-person | |  |  |  |  |  |  |
|  | Individual | -.36*** | -.32*** | -.28*** | -.28*** | -.27*** | -.34*** |
|  | Simultaneous | -.23*** | -.16*** | -.11*** | -.12*** | -.08** | -.24*** |

*Note.* “Individual” indicates that contentment, cheerfulness, and tranquility were examined individually. “Simultaneous” indicates that the three emotions were examined simultaneously. At the between-person, “individual” associations were examined using Pearson correlations, and “simultaneous” associations were computed using the standardized regression coefficients. The within-person associations were examined using standardized coefficients from hierarchical linear models, and all emotion scores were group-mean centered.

**Table S4**

*The associations between brokenness, exhaustion, and pleasurable emotions*

|  |  |  |  | Brokenness |  |  | Exhaustion |  |
| --- | --- | --- | --- | --- | --- | --- | --- | --- |
|  |  |  | cheer | content | tranquil | cheer | content | tranquil |
| Between-person | | |  |  |  |  |  |  |
|  |  | individual | -.24*** | -.35*** | -.34*** | -.30*** | -.37*** | -.43*** |
|  |  | simultaneous | .23* | -.38*** | -.21* | .11 | -.15 | -.39*** |
| Within-person | | |  |  |  |  |  |  |
|  |  | individual | -.22*** | -.24*** | -.23*** | -.30*** | -.26*** | -.30*** |
|  |  | simultaneous | -.09** | -.14*** | -.15*** | -.20*** | -.04 | -.20*** |

*Note.* “Individual” indicates that the three emotions were examined individually. “Simultaneous” indicates that the three emotions were examined simultaneously. At the between-person, “individual” associations were examined using Pearson correlations, and the “simultaneous” associations were examined using standardized regression coefficients. The within-person associations were examined using hierarchical linear models, and all emotion scores were group-mean centered.

**Table S5**

*The associations between brokenness, exhaustion, and psychological distress*

|  |  | Depression | Worry |
| --- | --- | --- | --- |
| Brokenness | |  |  |
|  | Between-Person | .80*** | .71*** |
|  |  |  |  |
|  | Within-Person | .38*** | .33*** |
|  |  |  |  |
| Exhaustion | |  |  |
|  | Between-Person | .74*** | .66*** |
|  |  |  |  |
|  | Within-Person | .39*** | .34*** |

*Note.* The between-person associations were computed using Pearson correlations. The within-person coefficients were computed using standardized hierarchical linear regression coefficients, and brokenness and exhaustion scores were group-mean centered.

**Table S6**

*The associations between contentment and depressive symptoms in the MIDUS sample, with and without the item “felt so sad that nothing would cheer you up”*

|  | *MIDUS* (n = 782) Depressive Symptoms | | | | *MIDUS* (n = 782) Adjusted Depressive Symptoms | | | |
| --- | --- | --- | --- | --- | --- | --- | --- | --- |
|  | Estimate | SE | *z* | *p* | Estimate | SE | *z* | *p* |
|  | Between-Person Associations | | | | | | | |
| Contentment | -0.26 | 0.03 | -10.39 | <.001 | -0.25 | 0.02 | -10.35 | <.001 |
| Tranquility | -0.23 | 0.02 | -9.38 | <.001 | -0.22 | 0.02 | -9.35 | <.001 |
| Cheer | -0.23 | 0.02 | -9.36 | <.001 | -0.22 | 0.02 | -9.39 | <.001 |
|  | Concurrent Within-Person Associations | | | | | | | |
| Contentment | -0.13 | 0.01 | -19.29 | <.001 | -0.10 | 0.02 | -5.88 | <.001 |
| Tranquility | -0.12 | 0.01 | -17.47 | <.001 | -0.11 | 0.02 | -6.12 | <.001 |
| Cheer | -0.12 | 0.01 | -18.16 | <.001 | -0.09 | 0.02 | -5.73 | <.001 |
|  | Prospective Within-Person Associations | | | | | | | |
| Contentment | -0.06 | 0.02 | -2.82 | .005 | -0.05 | 0.02 | -2.68 | .01 |
| Tranquility | 0.01 | 0.02 | 0.31 | .76 | 0.01 | 0.02 | 0.47 | .64 |
| Cheer | -0.01 | 0.02 | -0.69 | .49 | -0.01 | 0.02 | -0.66 | .51 |

*Note*. MIDUS Depressive Symptoms were identified using the items “felt worthless,” “felt so sad that nothing would cheer you up,” “felt like everything was an effort,” and “felt hopeless.” MIDUS Adjusted Depressive Symptoms were identified using the items “felt worthless,” “felt like everything was an effort,” and “felt hopeless.”
